# Post-transcriptional Modulation of Sphingosine-1-Phosphate Receptor 1 by miR-19a Affects Cardiovascular Development in Zebrafish

**DOI:** 10.3389/fcell.2018.00058

**Published:** 2018-06-05

**Authors:** Elena Guzzolino, Elena Chiavacci, Neha Ahuja, Laura Mariani, Monica Evangelista, Chiara Ippolito, Milena Rizzo, Deborah Garrity, Federico Cremisi, Letizia Pitto

**Affiliations:** ^1^Institute of Clinical Physiology, National Research Council, Pisa, Italy; ^2^Institute of Life Sciences, Scuola Superiore Sant'Anna, Pisa, Italy; ^3^Department of Biology, Center for Cardiovascular Research, Colorado State University, Fort Collins, CO, United States; ^4^Department of Clinical and Experimental Medicine, University of Pisa, Pisa, Italy; ^5^Tumour Institute of Tuscany, Florence, Italy; ^6^Scuola Normale Superiore di Pisa, Pisa, Italy

**Keywords:** sphingosine-1-phosphate receptor 1, microRNA, zebrafish, cardiovascular development, Holt Oram syndrome

## Abstract

Sphingosine-1-phosphate is a bioactive lipid and a signaling molecule integrated into many physiological systems such as differentiation, proliferation and migration. In mammals S1P acts through binding to a family of five trans-membrane, G-protein coupled receptors (S1PRs) whose complex role has not been completely elucidated. In this study we use zebrafish, in which seven *s1prs* have been identified, to investigate the role of *s1pr1*. In mammals *S1PR1* is the most highly expressed S1P receptor in the developing heart and regulates vascular development, but in zebrafish the data concerning its role are contradictory. Here we show that overexpression of zebrafish *s1pr1* affects both vascular and cardiac development. Moreover we demonstrate that *s1pr1* expression is strongly repressed by miR-19a during the early phases of zebrafish development. In line with this observation and with a recent study showing that miR-19a is downregulated in a zebrafish Holt-Oram model, we now demonstrate that *s1pr1* is upregulated in *heartstring* hearts. Next we investigated whether defects induced by *s1pr1* upregulation might contribute to the morphological alterations caused by Tbx5 depletion. We show that downregulation of *s1pr1* is able to partially rescue cardiac and fin defects induced by Tbx5 depletion. Taken together, these data support a role for *s1pr1* in zebrafish cardiovascular development, suggest the involvement of this receptor in the Tbx5 regulatory circuitry, and further support the crucial role of microRNAs in early phase of zebrafish development.

## Introduction

Sphingosine-1-phosphate (S1P) is a biologically active lysophospholipid with crucial role for membrane structure and function in eukaryotes. In vertebrates, S1P is found in the extracellular environment and interacts with cell-surface receptors to regulate an array of cellular responses, including cellular differentiation, proliferation, migration, cytoskeletal reorganization and apoptosis (Chun et al., 2002; Blaho and Hla, 2011). In particular, S1P action is implicated in the regulation of numerous cardiovascular processes including angiogenesis, vascular permeability, arteriogenesis, cardiac function, vascular development, and vascular tone (Levade et al., 2001; Allende and Proia, 2002; Alewijnse et al., 2004). Moreover, the involvement of S1P in mouse limb development (Chae et al., 2004) and neurogenesis (Mizugishi et al., 2005) has been also reported. After release from cells in response to various cellular stimuli, S1P acts in an autocrine and paracrine manner through its cell surface receptors. S1P receptors (S1PRs) are G protein-coupled receptors (GPCRs) critical for S1P action. In mammals, five *S1pr*s (*S1pr1–S1pr5*) have been identified. Three S1P receptor subtypes (*S1pr1,2,3*) are expressed in the adult cardiovascular system each with a unique pattern of expression (Alewijnse et al., 2004; Means and Brown, 2009).

In mammals, *S1pr1* is the most highly expressed S1P receptor in the cardiac myocytes in the developing heart; it also regulates vascular development in coordination with *S1pr2* and *S1pr3*. *S1pr1* knockout (KO) mice show intrauterine lethality between E12.5 and E14.5 because of severe hemorrhage (Liu et al., 2000). Recently analysis of S1pr1 LacZ knockin embryos revealed that S1P signaling via S1P1 in cardiomyocytes plays a previously unknown and necessary role in heart development in mice (Clay et al., 2016).

In zebrafish the *s1pr2* (*miles apart*) gene has been shown to play a crucial role in heart development by affecting the migration of myocardial precursor cells to the ventral midline of the embryo where they assemble into the heart tube. In accord with this function, deletion of *s1pr2* results in *cardia bifida* (Kupperman et al., 2000). In contrast, data concerning the *s1pr1* function in zebrafish are rather controversial: several papers in recent years highlighted the *s1pr1* role in vascular development and in controlling the venous endothelial barrier integrity, similar to the role played by this receptor in mouse (Ben Shoham et al., 2012; Gaengel et al., 2012; Tobia et al., 2012; Mendelson et al., 2013). All these data were obtained by morpholino-mediated loss of function experiments. However a recent paper by Hisano et al. analyzed all of the available *s1pr* zebrafish mutants generated by TALEN-mediated frameshift mutations. They demonstrated that none of the *s1pr* mutants showed developmental defects with the exception of *s1pr2* mutant which exhibits embryonic lethality arising from its cardiac defect (Hisano et al., 2015). These data suggest a previously unrevealed redundancy in functions of the S1P receptor-mediated signaling in zebrafish similarly to the partially overlapping expression of S1P receptors observed in mouse (Means and Brown, 2009).

In this study, to further investigate the role of *s1pr1* in zebrafish and to work around the redundancy problem, we overexpressed this receptor during early developmental stages. Our data support the involvement of *s1pr1* in cardiovascular development. Importantly, we show that *s1pr1* during the early zebrafish developmental stages is controlled by miR-19a and as such might be part of the Tbx5/miR-19a regulatory circuit affecting heart development.

## Materials and methods

### Reagents

Mature dre-miR19a-3p mimic (F 5′-UGUGCAAAUCUAUGCAAAACUGAUU-3′ and R 5′-UCAGUUUUGCAUAGAUUUGCUAAUU-3′) and a miR-Ct (F 5′-CUCUAGGUUAAACUCCUGGUU-3′ and R 5′-AACCAGGAGUUUAACCUAAUGUU-3′) were synthesized by GenePharma (Shanghai, China). Tbx5a morpholino(5′-GAAAGGTGTCTTCACTGTCCGCCAT-3′, Chiavacci et al., 2012) and *s1pr1* morpholino (5′-AGTGTCTGGCGATTAGGTCATCCAT-3′, Mendelson et al., 2013) were synthesized by Gene Tools (LLC USA.). QuikChange II XL site-directed mutagenesis kit (Agilent), α-minimal Essential Medium (Invitrogen, Life Technologies Italia, Monza, Italy); Polyfect, miRNeasy Mini Kit, miScript Reverse Transcription kit and Quantitec Reverse Transcription kit, (QIAGEN, Milan, Italy); pGEMTeasy vector andDual-Luciferase Reporter Assay System (PROMEGA); pCS2^+^ vector (Addgene); zebrafish diet (SDS, Dietex,France); mMESSAGEmMACHINE SP6 transcription Kit (Thermo Fisher); (R)-3-amino-4-(3-hexylphenylamino)-4-oxobutylphosphonic acid trifluoroacetate (W146) (Avanti, Polar Lipid. Inc. USA).

### Zebrafish lines

Wild-type AB, *Tg(flk1:EGFP)*,*Tg(Myl7:EGFP)* and the Tbx5a^s296^ mutant lines were used in these studies. Zebrafish were raised and maintained under standard laboratory conditions (Westerfileld M zebrafish book) in Zebrafish Housing Systems (Tecniplast, Varese, Italy). The local ethics committee approved animal studies and all procedures conformed to the essential ethical rules and the current applicable legislation. Adult zebrafish were bred under standard conditions and embryos obtained by natural spawning and incubated at 28.5°C in E3 medium (Westerfield M. zebrafish book). They were further staged and fixed at specific time-points as described by Kimmel (Kimmel et al., 1995). When animals need to be euthanized, an overdose of tricaine methane sulfonate (200–300 mg l^−1^) by prolonged immersion was used, which is a well-established humane method.

### Zebrafish microinjection

Transgenic *Tg(Myl7:eGFP)* or *Tg(flk1:EGFP)* zebrafish embryos were injected at the 1 cell stage with a constant injection volume (~1 nl, confirmed by volume analysis) using a microinjector made by Tritech Research (Los Angeles USA).

### Cells culture and transfection

HEK-293 cells were grown in DMEM + 10% FBS, 2μg/ml L-glutamine and 50 μg/ml streptomycin at 37°C in a humidified atmosphere containing 6% CO_2_. Cells were seeded at a density of 1.5 × 10^5^ cells per well in 12 well dishes and grown for 24 h. Twenty-four hours later, cells were transfected using Polyfect (Qiagen) as transfectant, according to the manufacturer's recommendations. In each transfection 100 ng/μl of the s1pr1-3′UTR-*wt* construct, the s1pr1-3′UTR-mut, containing the mutation in the miR-19a binding site, or the pGLU empty vector as control were co-trasfected with increasing doses of duplex si-miRNA19a in the presence of 100 ng/μl of *Renilla* expressing plasmid as internal standard. In each transfection the total amount of the transfected si-miRNA was kept constant by adding a scrambled miRNA (miR-Ct) to the specific miR-19a to obtain 80 ng si-miRNA concentration. After 24 h at 37°C cells were washed with PBS for two times and processed for the Luciferase assay (Verduci et al., 2010).

### *s1pr1* cloning and *in vitro* transcription

The *s1pr1* CDS and full-length clones were obtained using cDNA generated from total RNA of 48 hpf wild type zebrafish embryos. The *s1pr1* CDS was PCR amplified using the following primers: F: 5′-ATGGATGACCTAATCGC-3′ and R: 5′-ACGACAAAGTTCACGAATAGTC-3′. To generate the full-length clone including the 854 bp of the 3′UTR, a different reverse primer was used R-full: 5′-GAACAGGGACAAAACTGGCTC-3′. Following ligation into the pGEM T-easy vector, the inserts were subcloned into the PCS2 vector and verified by sequencing. The inserts were linearized with NotI and capped mRNAs were generated using the mMESSAGEmMACHINE SP6 transcription Kit. The same kit was used to *in vitro* transcribe the RFP CDS from the NotI linearized pCS2+ vector.

The *s1pr1* 3′UTR was PCR amplified using the following primers: F: 5′-*GGTACC*TCTTCTTCTTAAAGC-3′ (where italics indicates the bases added to generate the restriction site KpnI) and R: 5′-GAACAGGGACAAAACTGGCTC-3′. Following ligation into the pGEM T-easy vector, the insert was excised using KpnI/SalI, subcloned into KpnI/XhoI of the pGLU Dual-luciferase reporter plasmid (Poliseno et al., 2010) and verified by sequencing. The mutated version of the *s1pr1* 3′UTR (*s1pr1*-3′UTR-mut) was generated utilizing the *s1pr1*-3′UTR *wt* plasmid as template and modifying the miR-19a seed binding site using the QuikChange II XL site-directed mutagenesis kit. To generate the GFP sensor reporter vector for zebrafish injection the *s1pr1* 3′UTR was excised from pGLU-s1pr1-3′UTR vector using XbaI and the insert was subcloned into XbaI linearized pCS2-GFP vector. The correct insert orientation was checked by restriction digestion analysis.

### *s1pr1* chemical inhibition in zebrafish embryos

W146 was dissolved in a solution of 20% 2-hydroxypropyl-beta-cyclodextrin (Tarrason et al., 2011) and 50 mM NaCO2 at the stock concentration of 100 μM/ml. 24 hpf non-injected and Tbx5 morphant embryos were exposed to W146 at the following concentrations: 0, 0.00025, 0.005, 0.05, 0.5, and 1 μg/ml. We started from 0.00025 μg/ml since the 0.005 μg/ml is the concentration sufficient to cause vessel defects when injected intravenously in mouse (Tarrason et al., 2011) but we were not able to use a dose higher than 1 μg/ml due to solubilization problem. The quantity of solvent added to E3 medium was kept constant among the different treatments. Embryos were scored at 72 hpf for heart phenotypes, fin phenotypes, as well as heart rate. For heart rate, each fish was scored three times. Heart beats were counted for either 10 or 20 s, and average heartbeats/min was calculated. Statistics were run on the average heartbeats/min of the three trials.

### Dual-luciferase reporter assay and *in vivo* GFP assay

Luciferase activity was measured 24 h after transfection using the Dual-Luciferase Reporter Assay system. HEK 293 cells after PBS washing were extracted by addiction of 100 μl Passive Lysis Buffer 1x (Promega) in each dish. After 5 min at room temperature the plate was put at −80°C for 10 min. The contents of each dish was collected and centrifuged for 10 min at 4°C. The obtained supernatant was immediately assayed or stored at −80°C. Firefly luciferase activity was normalized to *Renilla* activity for each transfected dish. Assays were performed in three independent experiments.

For the *in vivo* sensor assay, approximately 500 pg of the pCS2-GFP mRNA was coinjected with either si-miR-19a or control mimic (10 μM) into single-cell stage (1 nl injection volume) embryos. 24 hpf after microinjection embryos were analyzed by fluorescent microscopy.

### Imaging

Staining was observed with Leica M80 microscope and images were acquired with Nikon DS-Fi1 camera and NIS-Elements F 3.0 software. For fluorescence microscopy Leica DM IL microscope and Nikon YFL microscope both equipped with CoolSnap CF camera (Photometric) were used. Images were processed with Gimp-2.6 or ImageJ software.

Confocal imaging was performed with Leica TCS SP8 confocal laser scanning microscope (Leica Microsystems, Mannheim, Germany) equipped with Leica Application Suite (LAS) X software. The Z-stack function scanned the organisms along the “z” dimension. All confocal frames were taken with a good level of resolution along with a low scanning speed and a specific setting to visualize the green signal (format 1,024 × 1,024 dpi resolution).

### Heart dissection

Zebrafish hearts were dissected with physical pressure using a glass pipette according to Singleman and Holtzman (2011). Embryos were previously anesthetized with tricaine (0.04%) and physical heart dissection was performed in L15 medium with the addition of 10% sheep/bovine serum. After the dissection about 25 hearts for each lines were extracted. Three extractions from 3 different clutches were performed.

### RNA extraction and quantitative real time RT-PCR and digital droplet PCR

Total RNA was extracted using the miRNeasy Mini kit (Qiagen). RNA was quantified using a NanoDrop-1000 spectrophotometer and quality was monitored by the Agilent 2100 Bioanalyser (Agilent Technologies, Santa Clara, CA). cDNA was reverse transcribed using miScript Reverse Transcription kit (for miRNA analysis) and Quantitec Reverse Transcription kit (for gene analysis). Real-time PCR (qRT-PCR) was carried out using QuantiFast SYBR Green kit with Rotor gene (Qiagen). Relative quantification was performed as previously described (Chiavacci et al., 2015). Elongation factor α (*ef1*α) was used as the reference gene. For Digital Droplet PCR the QX200 droplet digital PCR system (BioRad) was used following the manufacturer's instruction. After PCR, read-out of positive vs. negative droplets was performed with the droplet reader and the absolute quantification of PCR target was analyzed using QuantaSoft software (BioRad). Absolute readouts were normalized to the amount of *ef1a* present in each sample. The list of primers used is reported in Table 1.

**Table 1:** List of oligonucleotides used in this study.

**Gene**	**Forward primer**	**Reverse primer**
*ef1α*	CTGGAGGCCAGCTCAAACAT	ATCAAGAAGAGTAGTACCGCTAGCATTAC
*nppa*	CAACATGGTCAAGCTCAA	GGCTCTCTCTGATGCCTCTTC
*hand2*	AAGGCGAAAGAAGGAAATGAA	GCCAACCAGTTCTCCCTTTA
*nkx2.5*	TGACACATTTGAAGACAAAGAGAAA	TCCTCCTCTTCCTCTGCTTG
*mef2caA*	GAAACACAGGAGGTCTGATGG	GTGGTTTCCGTACCCGTTT
*mef2aa*	GGGGACCACGGAGAAAAA	TGGCTTTCAATGCCTTCTCT
*cx43*	TCGCGTACTTGGATTTGGTGA	CCTTGTCAAGAAGCCTTCCCA
*bmpr1aa*	GCGTCAGCTTTTGTTCATCA	TGATCAGGATTCTGACCTGCT
*cyp26b1*	GCCAACTCAATAGGAGACATCC	CCAGAGCCTCATGGCTAAAAA
*gata4*	TCGCACTTCGACAGCTCCGTA	GACATCGCCCCGCAGTTCACA
*s1pr1*	TGTCAGACCCTCACCTGCT	TTCATGGCAGAGTTGAGCAC
*s1pr2*	CACGCGCTTCTTCTCTCC	CAGCCCGAAGTCACGTCT
*s1pr3a*	CATACCGCAGAGAACAGCAAC	CTGACTTGGCTGCACCACTA
*s1pr4*	AACCGAAGAACGGCAAAAA	CGCTTGACGCAGATAAACAA
*s1pr5a*	CATGCCGTTTCTGGATTGTA	AGGCCTTCCAGCCTGTGT
*s1pr5b*	AGAACCTGACGGTCCTGCT	GGTCCGATAGTGCCAGGTT

### Whole mount *in situ* hybridization

Whole mount *In Situ* Hybridization (ISH) was performed as previously described (Chiavacci et al., 2012).

### Statistical analysis

Data were analyzed using GraphPad Prism (GraphPad Software, San Diego, CA USA). Statistical differences were determined by unpaired *t*-test, and Fisher's test with values of *P* < 0.05 were considered statistically significant. Each experimental point in the graph represents the mean ± SE of at least three independent experiments.

## Results

### *s1pr1* overexpression affects heart, fin and vascular development in a dose-dependant way

**Figure 1:**
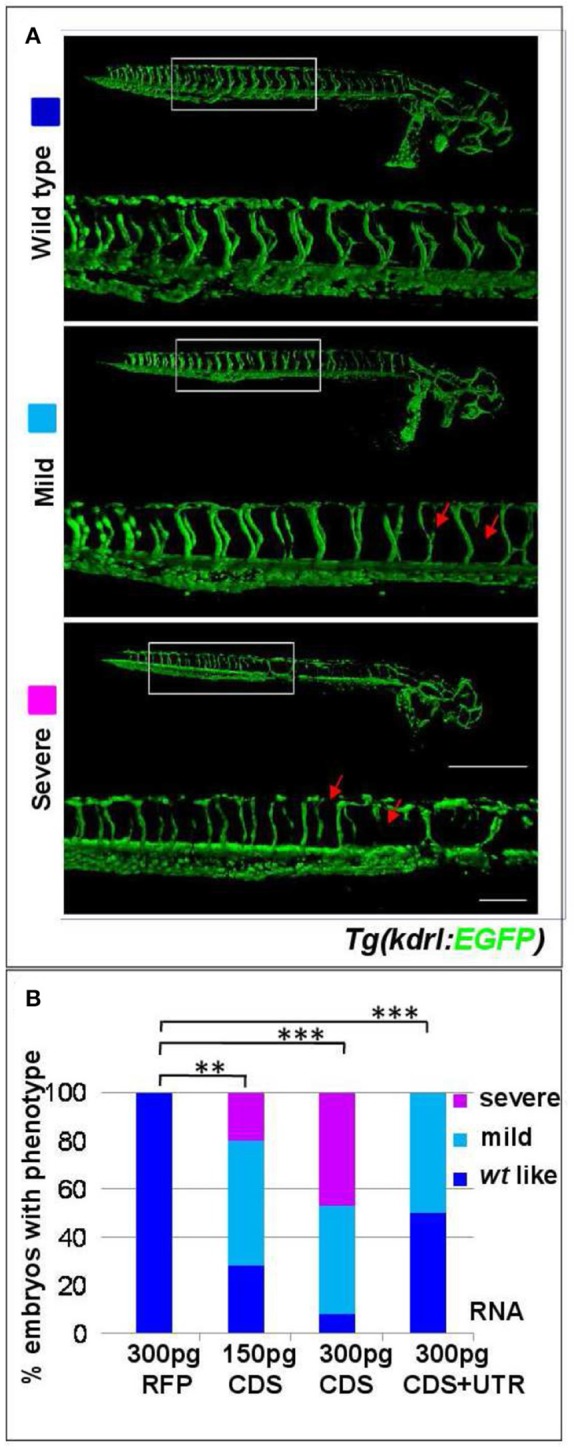
*s1pr1* overexpression affects vascular development. **(A)** Confocal microscopy of representative 48 hpf *Tg(kdrl:EGFP)*^*s843*^ embryos showing vascular defects of different severity. The *Tg(kdrl:EGFP)*^*s843*^ embryos were injected with *in vitro* transcribed and capped mRNA of the full-length (CDS+UTR) or the CDS sequence of *s1pr1* at the reported doses (pg). The total numbers of embryos analyzed were as follows: 300 pg RFP CDS injected *n* = 20; 150 pg s1pr1 CDS injected *n* = 22; 300 pg s1pr1 CDS injected *n* = 38; 300 pg s1pr1 CDS+UTR injected *n* = 15. Red arrows indicate examples of shorter and incomplete ISVs. Scale bar = 500 μm (zoom 100 μm). **(B)** Blinded quantification of the abnormal vascular phenotypes showed in **(A)**. ^**^P < 0.001, ^***^P < 0.0001.

The coding sequence (CDS) of *s1pr1* was cloned and *in vitro* transcribed. 150 and 300 pg of the obtained *s1pr1* CDS RNA were injected in *Tg(kdrl:EGFP)*^*s843*^ transgenic zebrafish embryos in which EGFP expression is driven by the promoter of the pan-endothelial marker *vegfr-2* (Beis et al., 2005). As control, the RNA of Red Fluorescent Protein (RFP) obtained from *in vitro* transcription of the pCS2+ vector was used. As already reported, *s1pr1* overexpressing (S1up) embryos show alteration in the intersegmental vessel (ISV) formation and in the caudal vein plexus (CVP) (Mendelson et al., 2013). In S1up embryos, the ISV are shorter and truncated compared to ISVs in the control (Ct) embryos and the area of CVP is more compact compared to the honeycomb-like morphology which characterizes the Ct embryos. We included in the mild phenotypes embryos showing only shorter ISV and almost normal CVP while we considered as severe embryos with truncated ISV and more compact CVP (Figure 1B). Both ISV and CVP defects show *s1pr1* dose-dependent increase in severity (Figures 1A,B).

**Figure 2:**
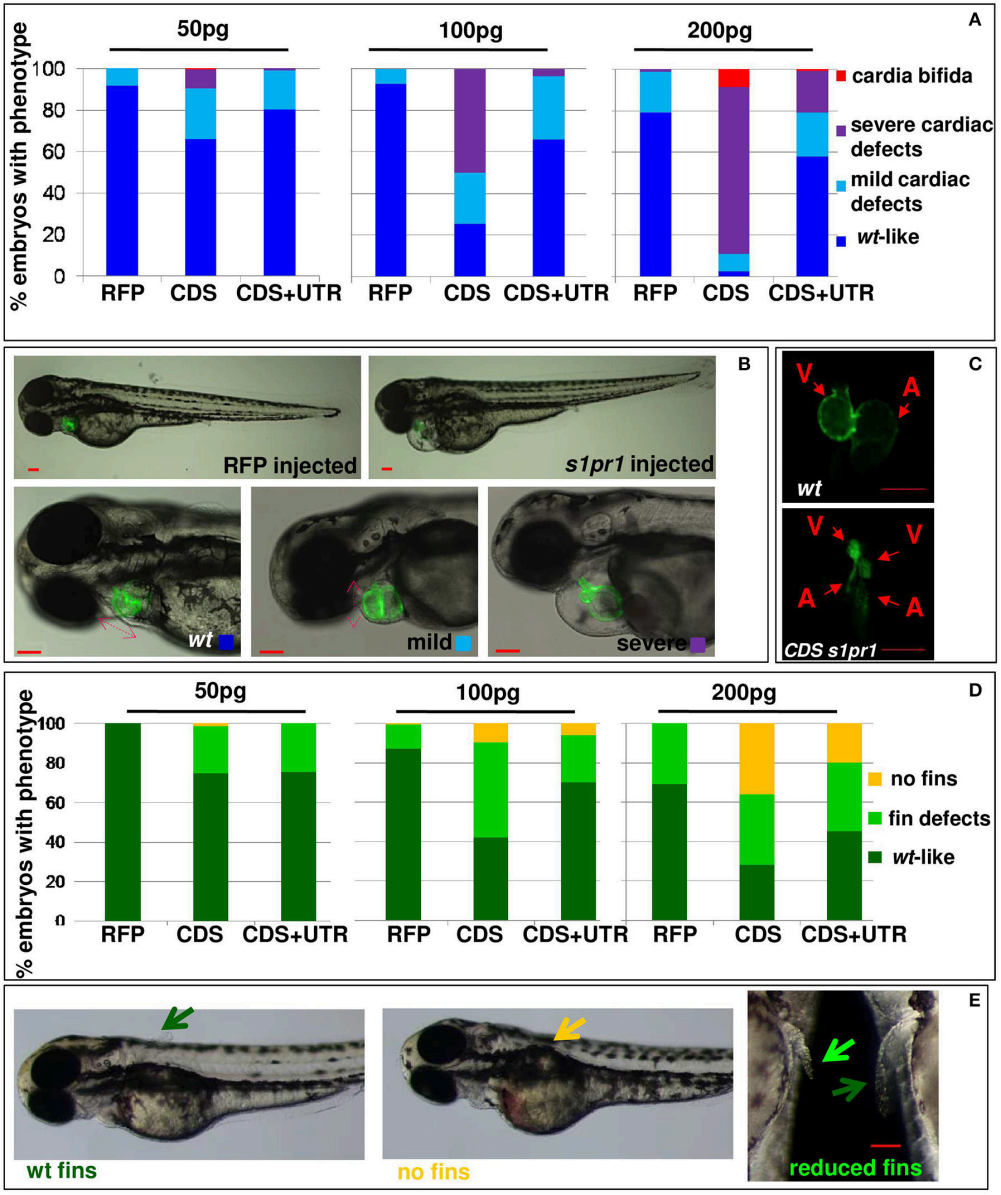
*s1pr1* overexpression affects heart and fin development in a dose-dependent way. Analysis of 72 hpf *Tg(Myl7:EGFP)* embryos injected with CDS or CDS+UTR mRNA of *s1pr1*at the reported doses (pg). The percentage of embryos with the indicated heart **(A)** or pectoral fin **(D)** defects was averaged across multiple independent experiments carried out in double blind. The total number of analyzed embryos were as follows: RFP mRNA injected *n* = 185 (50 pg), *n* = 138 (100 pg), *n* = 89 (200 pg); *s1pr1*CDS mRNA injected *n* = 195 (50 pg), *n* = 199 (100 pg), *n* = 106 (200 pg); *s1pr1* CDS+UTR mRNA injected *n* = 214 (50 pg), *n* = 198 (100 pg), *n* = 96 (200 pg). For all tested doses, differences between control and *s1pr1* injected embryos were significant (Fisher's test *P* < 0.0001) with the only exception of the 50 pg mRNA CDS+UTR which was not significant. **(B,C,E)** Images representative of the different phenotypes. In **(B)**, the red dashed arrows emphasize the valve orientation as indicator of correct looping. In **(C)** an example of *cardia bifida* phenotype is shown. In **(E)** the yellow arrow indicates the absence of fins, the green arrows indicate WT fins and the pale green arrow points to an example of defective fin. V, ventricle; a, atrium. Red scale bar = 100 μm.

In order to investigate whether *s1pr1* overexpression also affects cardiac development, *s1pr1* CDS mRNA was injected in *Tg(myl7:EGFP)* embryos in which cardiomyocytes are marked with green fluorescence (Huang et al., 2003). Increasing doses of *s1pr1* raised the frequency and severity of cardiac defects (Figures 2A,B). In the less severe phenotypes, only cardiac looping was affected while in the most severe phenotypes also the chamber shape showed alteration and in particular the ventricle was smaller and irregular and the atrium was dilated (Figure 2B). In a few cases, *cardia bifida* was evident as two small deformed beating hearts located on either side of the midline (Figure 2C). Interestingly, the occurrence of defective or absent pectoral fins paralleled the *s1pr1* dose-dependent increase of cardiac defects (Figures 2D,E). Embryos injected with the same dosage of RFP mRNA showed no apparent phenotype (Figure 2B). On the contrary, downregulation of *s1pr1* by morpholino injection, although strongly affecting vascular morphology as largely demonstrated in several papers (Ben Shoham et al., 2012; Gaengel et al., 2012; Tobia et al., 2012; Mendelson et al., 2013), has a limited effect on heart development. The most frequent cardiac alteration we observed was an enlargement of atrial size, which occurred in about 20% of injected embryos (Figure S1).

To investigate the molecular consequences of increased cardiac expression of *s1pr1*, hearts were dissected from 48 hpf embryos previously injected with CDS mRNA of *s1pr1* or RFP as control. Total RNA was extracted from pools of 20–30 hearts in three different experiments and first of all we verified whether upregulation of *s1pr1* might impact the cardiac level of the other members of the s1p receptor family. We analyzed by Q-RT PCR the expression of *s1pr2, s1pr3a, s1pr3b, s1pr4, s1pr5a*, and *s1pr5b* (Data Sheet 1) but only *s1pr2* and *s1pr5a* showed an expression level comparable to *s1pr1* in the RFP injected hearts. All the other receptors were not detectable at the cardiac level both in RFP and in *s1pr1* injected hearts. As a consequence of *s1pr1* overexpression we observed a significant but small upregulation of *s1pr2* while the level of *s1pr5a* was not changed (Figure S2).

**Figure 3:**
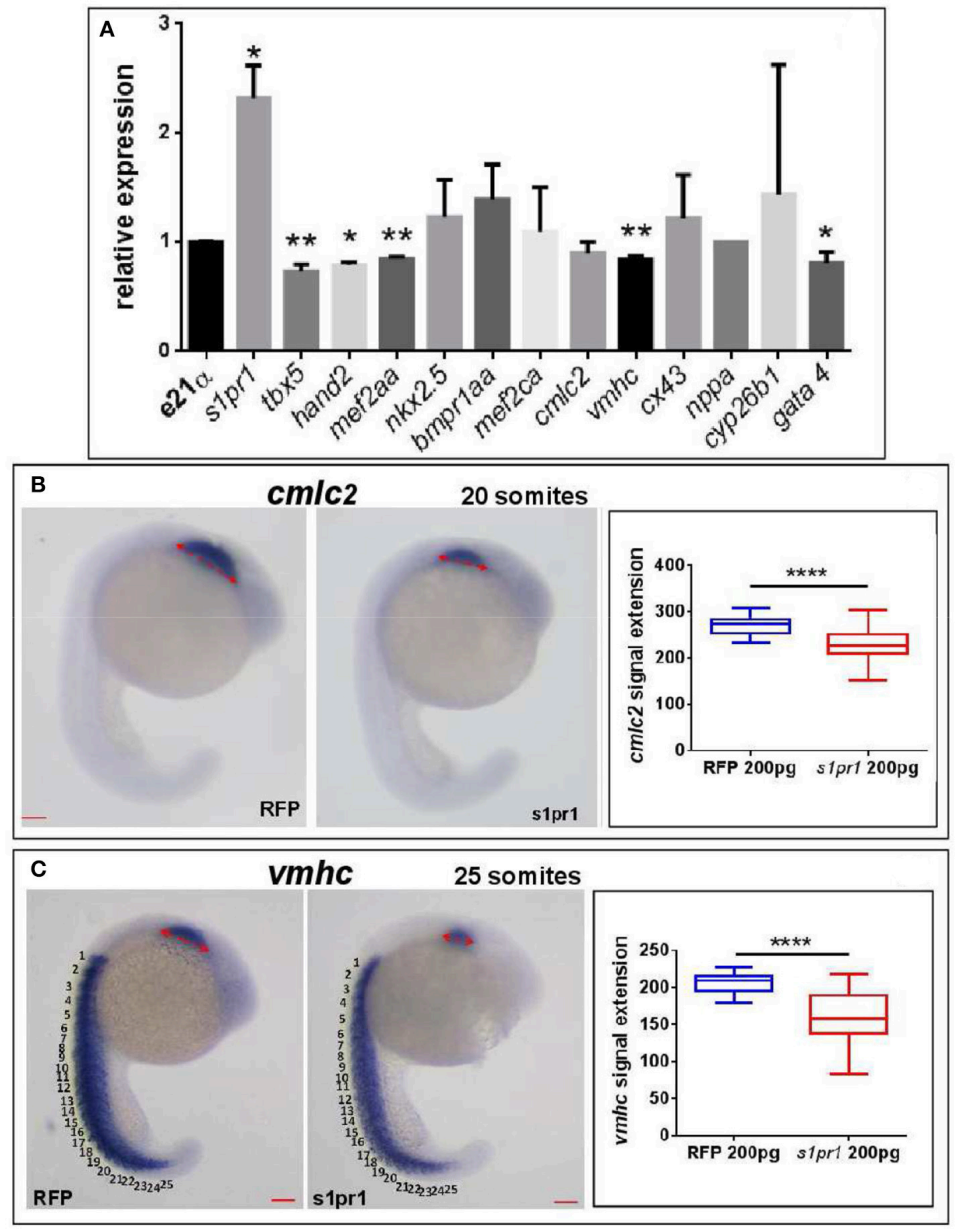
*s1pr1* overexpression significantly affects the expression of cardiac precursors and some cardiac regulators. One cell stage embryos were microinjected with 200 ng of the CDS mRNA of *s1pr1* or *RFP*. **(A)** 48 h after microinjection 20–30 hearts for each experiment were dissected as described in Materials and Methods section. Total RNA was extracted, reverse-transcribed and Q-RT PCR performed. Data were normalized using ef1α as internal standard and are relative to values of hearts from RFP injected embryos. Three different experiments starting from 3 different clutches were used for this analysis.*P < 0.05, **P < 0.001. **(B,C)** cmlc2 **(B)** and vmhc **(C)** ISH were performed on 20 **(A)** and *25* **(B)** somite stage embryos. Left side, examples of ISH: double head arrows indicate the extension of the hybridization signals. Scale bar = 100 μm. Right side shows the quantifications of the hybridization signals: the arrow lengths in μm are reported. About 40 embryos were analyzed for each experiment. ****P < 0.0001.

Next we quantified by Q-RT PCR several cardiac markers which characterize cellular determination, patterning and differentiation of cardiac phases (Data Sheet 1). We observed a small but significant downregulation of some important cardiac regulators such as *tbx5, hand2, mef2AA*, and *gata4* (Figure 3A). Following gastrulation, zebrafish, *gata4, hand2*, and *tbx5* are expressed bilaterally in portions of the Lateral Plate Mesoderm (Serbedzija et al., 1998; Reiter et al., 1999; Begemann and Ingham, 2000; Ruvinsky et al., 2000) and contribute to the especially intricate process of patterning of the LPM cardiogenic region.

Both *tbx5* and *hand2* are involved not only in zebrafish heart but are also expressed in the fin field and contribute to pectoral fin development (Yelon et al., 2000; Garrity et al., 2002; Parrie et al., 2013). Interestingly Hand2, Mef2AA, and Gata4 proteins are all Tbx5 interactors (Ghosh et al., 2009; Lu et al., 2016). Since the alteration of heart morphology might be a consequence of the vessel organization disruption (Auman et al., 2007; Dietrich et al., 2014), by *in situ* hybridization we investigated whether *s1pr1* overexpression might influence the myocardial precursor pool before heart tube formation. Figures 3B,C highlight a reduction of myocardial precursors, specifically ventricular primordium in the presence of an excess of *s1pr1* supporting a role of this receptor in the cardiac context.

These data support the hypothesis that *s1pr1* dysregulation in zebrafish heart might affect early stages of heart development.

### *s1pr1* is a direct target of miR-19a

During the last decade, several studies by our group and others clearly showed that microRNAs are critical components of the cardiogenic regulatory network, and play numerous roles in the growth, differentiation, and morphogenesis of the developing heart (van Rooij et al., 2008; Cordes and Srivastava, 2009; Chiavacci et al., 2012; Agostini et al., 2015; D'Aurizio et al., 2016). MicroRNAs exert their activity by preferentially binding to specific sites within the 3'-UTR of the mRNA target (Bartel, 2009).

Therefore we decided to more fully investigate the impact of *s1pr1* overexpression during the early stages of zebrafish development by overexpressing the full sequence of the *s1pr1* transcript spanning the CDS and 854 bp of the 3′UTR (CDS+UTR). We observed that the presence of the 3′UTR within the injected mRNA strongly decreases the impact of *s1pr1* overexpression on vascular and cardiac defects (Figures 1, 2 compare CDS values with CDS+UTR values). These results indicate that *s1pr1* might be negatively controlled at the post-transcriptional level at least during the first stages of zebrafish embryonic development.

In order to investigate whether *s1pr1* might be controlled by miRNAs we used Targetscan to identify potential miRNA targets in the zebrafish *s1pr1* 3′UTR. Several targets were identified by this algorithm; however, our attention focused on miR-19a for several reasons: we recently highlighted a role for miR-19a in a zebrafish model of Holt-Oram (HOS), a pathology affecting heart and upper limbs (pectoral fins) both in mammals and fishes (Chiavacci et al., 2015). miR-19, as a component of the miR-17-92 cluster, has been shown to be also involved in postnatal angiogenesis (Suarez et al., 2008) and to exert a pro angiogenic role in endothelial cell culture (Chamorro-Jorganes et al., 2016). Moreover, miR-19a,b are the miRNAs that obtain the highest score when matched to mouse and human *s1pr1* UTR target sequences, according to Targetscan (www.targetscan.org/vert).

**Figure 4:**
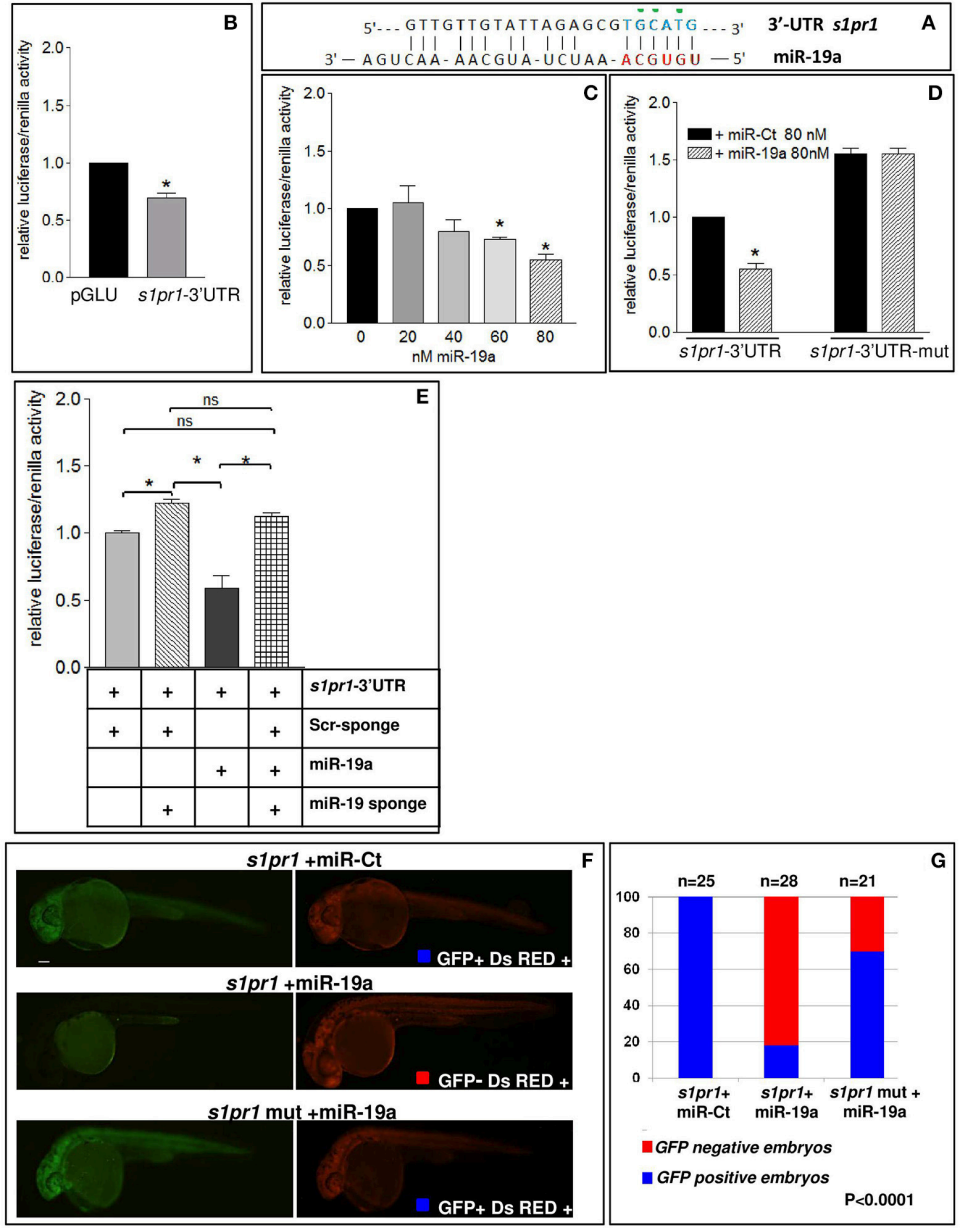
*s1pr1* is a direct target of miR-19a. **(A)** The computational alignment of a potential target site of miR-19a in *s1pr1* of *Danio rerio*. The green dots indicate the bases mutated to destroy the seed sequence binding in the s1pr1-3′UTRmut construct. **(B–E)** The 3′UTR of *dre-s1pr1* was cloned in the pGLU Dual-luciferase reporter plasmid (s1pr1-3′UTR) and transiently transfected into HK293 cells together with the Renilla luciferase pRL–TK vector as internal standard. The luciferase activity of s1pr1-3′UTR was compared: **(B)** with the luciferase activity of the empty vector; **(C)** in the presence of increasing amounts of miR-19a mimic; **(D)** with the luciferase activity of s1pr1-3′UTRmut (containing the s1pr1 3′UTR mutated in the seed match for miR-19a) in the presence of 80 nM of miR-19a mimic; **(E)** in the presence of 80 nM of miR-19a mimic and 500 pg of a sponge for miR-19a or 500 pg of a scrambled sponge. In each transfection the total amount of the transfected miRNA was kept constant by adding a scrambled miRNA (miR-Ct) to the specific miRNA to obtain 80 ng. *p < 0.05. **(F,G)** miR-19a negatively regulates the sensor s1pr1-GFP containing the *wt*3′UTR of *s1pr1* downstream the GFP sequence but it is inefficient on the mut s1pr1-GFP; **(F)** representative 24 hpf embryos injected with *wt*or mut sensor mRNAs, miR-19a or miR-Ct mimics and DsRED mRNA. Left, miR-19a presence reduces the embryo specific fluorescence in the presence of *wt*3′UTR of *s1pr1* and only the yolk autofluorescence is visible; right, DsRED expression is constant in all the experiment **(G)** quantification of the *in vivo* sensor assay. About 20 embryos were analyzed for each experiment.

In order to verify whether miR-19a is able to modulate *s1pr1* expression, the zebrafish *s1pr1* 3′UTR (GenBank: BC075741.1) was cloned downstream of the luciferase firefly reporter construct pGLU (Poliseno et al., 2010) creating the pGLU-*s1pr1*-3′UTR construct. HEK-293 cells were co-transfected with the pGLU-*s1pr1*-3′UTR vector plus increasing doses of miR-19a mimic (Mi-19a). As expected, the presence of the *s1pr1* 3′UTR sequence downstream of the luciferase coding sequence significantly decreased the reporter activity (Figure 4B). Additional increases in the amount of miR-19a produced further reductions in the relative luciferase activity in cells transfected with pGLU-*s1pr1*-3′UTR vector but not in cells transfected with the control pGLU-s1pr1-3′UTRmut, in which 3 bases within the seed match sequence of miR-19a binding site were mutated (Figures 4A,C,D). Co-transfection of Mi-19a and miR-17-92 cluster sponge (a mRNA containing 2 repeats of the full miR-17-92 cluster sequence) abolished the repressive effect exerted by this microRNA on the activity of the pGLU-*s1pr1*-3′UTR (Figure 4E). In line with these data, over-expression of miR-19a in zebrafish embryos significantly reduced the translational rate of a reporter construct (sensor), carrying GFP coding sequence upstream of *s1pr1*-3′UTR (Figure 4F). This reduction is almost erased if the mutated *s1pr1*-3′UTR is following the GFP sequence (Figure 4G).

These results indicate that *s1pr1* functions as a direct target of miR-19a.

### *s1pr1* and miR-19a functionally interact in the cardiac context

To further investigate the functional interaction between *s1pr1* and miR-19a in the cardiac context, we assessed whether the morphological alterations generated by *s1pr1* overexpression might be attenuated or exasperated by miR-19a increases or decreases, respectively. To modulate miR-19a levels, 1-cell stage *Tg(myl7:EGFP)* embryos were injected with Mi-19a or a specific morpholino against miR-19a (MO-19a) whose efficacy against miR-19a has been already shown (Chiavacci et al., 2015).

**Figure 5:**
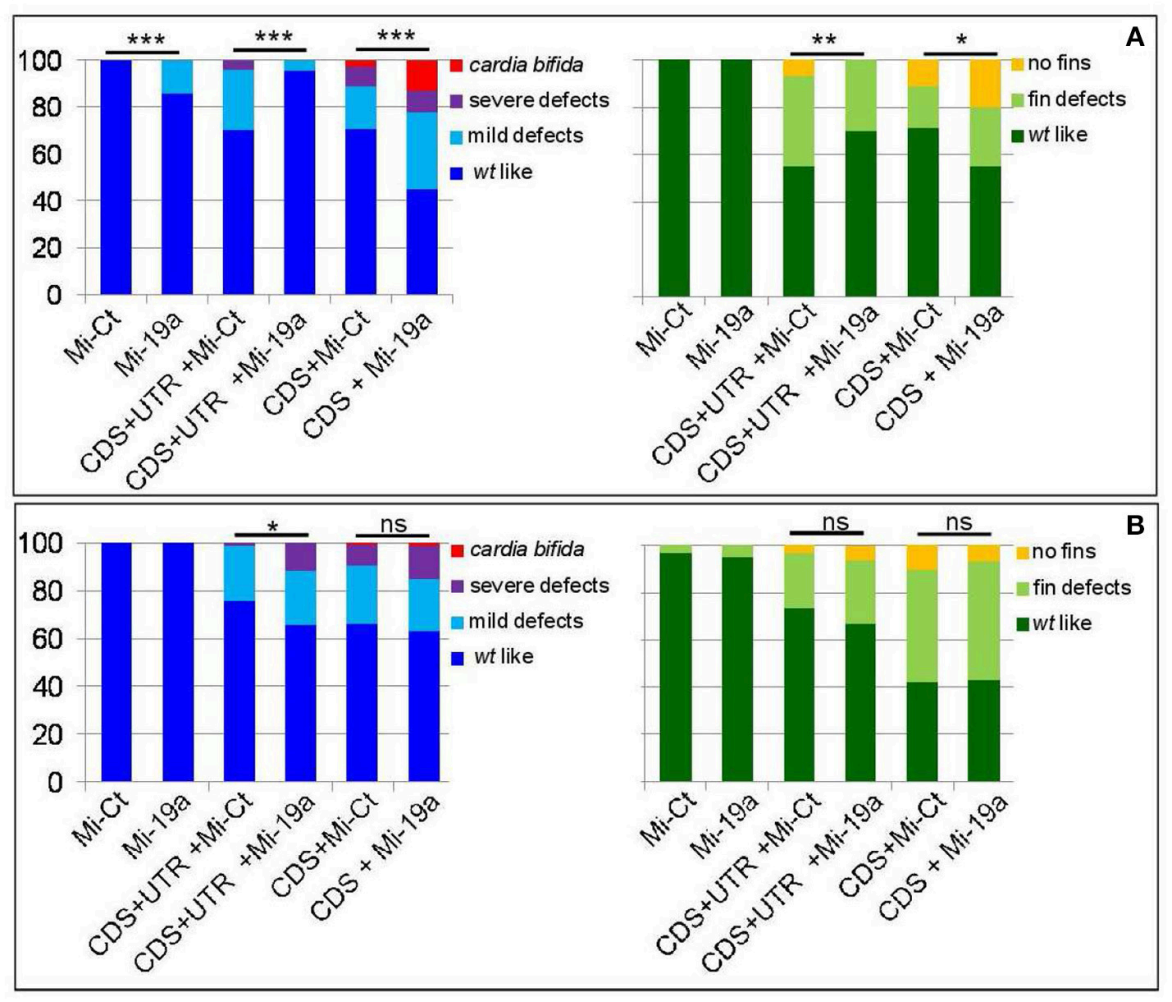
miR-19a modulation impacts the fin and cardiac defects induced by the overexpression of *s1pr1*. Analysis at 72 hpf of *Tg(Myl7:EGFP)* embryos injected with CDS (50 pg) or CDS+UTR (100 pg) mRNA of *s1pr1* in the presence of 0.5 ng of miR-19a (Mi-19) or miR-Ct (Mi-Ct) mimics **(A)** or in the presence of 8 ng of MO-19a or MO-Ct **(B)**. For comparison the phenotypes of embryos injected only with Mi-19a or Mi-Ct or with MO-19a or MO-Ct are presented. The percentage of embryos with the indicated heart (left) or pectoral fin (right) defects was averaged across multiple independent experiments carried out in double blind. The total number of analyzed embryos were as follows: Mi-Ct injected = 120; Mi-19a injected = 99; *s1pr1* CDS+UTR mRNA +Mi-Ct injected *n* = 133; *s1pr1* CDS+UTRmRNA + Mi-19a injected *n* = 142; *s1pr1* CDS mRNA + Mi-Ct injected *n* = 155; *s1pr1* CDS mRNA + Mi-19a injected *n* = 148; MO-Ct injected = 103; MO-19a injected *n* = 87; *s1pr1* CDS+UTR mRNA + MO-Ct injected *n* = 134; *s1pr1* CDS+UTR mRNA + MO-19a injected *n* = 156; *s1pr1* CDS mRNA + MO-Ct injected *n* = 72; *s1pr1* CDS mRNA +MO-19a injected *n* = 65. *p < 0.05,**P < 0.001, ***P < 0.0001. For the other statistical analysis: **(A)** left, comparisons vs. Mi-Ct (line 3 and 5 vs. 1) have both *P* < 0.0001, while comparisons vs. Mi-19 has *P* < 0.05 (line 4 vs. 2) and *P* < 0.0001(line 6 vs. 2); **(B)** left, all the comparisons (line 3 and 5 vs. 1 and line 4 and 6 vs. 2) have *P* < 0.0001; **(A,B)** right, all the comparisons (line 3 and 5 vs. 1 and line 4 and 6 vs. 2) have *P* < 0.0001.

As we previously showed (Chiavacci et al., 2015), the increase of miR-19a alone negatively impacts cardiac development but not fin morphology (Figure 5A). However injection of 0.5 ng of Mi-19a together with 100 pg of full *s1pr1* transcript significantly reduced the cardiac and fin defects caused by *s1pr1* overexpression. Conversely, the presence of Mi-19a exacerbates the cardiac and fin defects generated by the overexpression of *s1pr1* CDS lacking the relevant 3′UTR. Interestingly, co-injection of Mi-19a and *s1pr1* CDS resulted in a synergistic negative interaction: while injection of either 0.5 ng of Mi-19a or 50 pg of *s1pr1* CDS resulted respectively in 86 and 71% of *wt*-like hearts, the co-injection of Mi-19a and *s1pr1* CDS at the same concentrations produced only the 45% of embryos displaying a *wt*-like phenotype and strongly increased the percentage of embryos with both mild and severe cardiac defects (Figure 5A). Although a specific analysis of cardiac genes controlled by miR-19a is not described, it is interesting to note that miR-19a has been reported to downregulate mef2ca and mef2aa (Chiavacci et al., 2015). Therefore since our data indicate a negative impact of *s1pr1* on mef2aa (Figure 3A), the synergistic repression of both miR-19a and *s1pr1* on this gene which is highly expressed in zebrafish heart and involved in zebrafish heart function (Wang et al., 2005), might be one of the cause of the strong increase of cardiac defects observed in embryos co-injected with Mi-19a and *s1pr1* CDS.

Although high doses (10 ng) of MO-19a did not affect embryo morphology when injected alone (Chiavacci et al., 2015) and (Figure 5B), it increased the severity of cardiac defects caused by *s1pr1* overexpression. This effect was no longer detectable when only the CDS of *s1pr1* was co-injected with MO-19a (Figure 5B). The decrease of miR-19a level had no significant effects on the fin morphology (Figure 5B).

Overall these data support a role of miR-19a as regulator of *s1pr1* at least in the cardiac context.

### Downregulation of *s1pr1* partially rescues cardiac and fin defects induced by Tbx5 depletion in zebrafish HOS model

**Figure 6:**
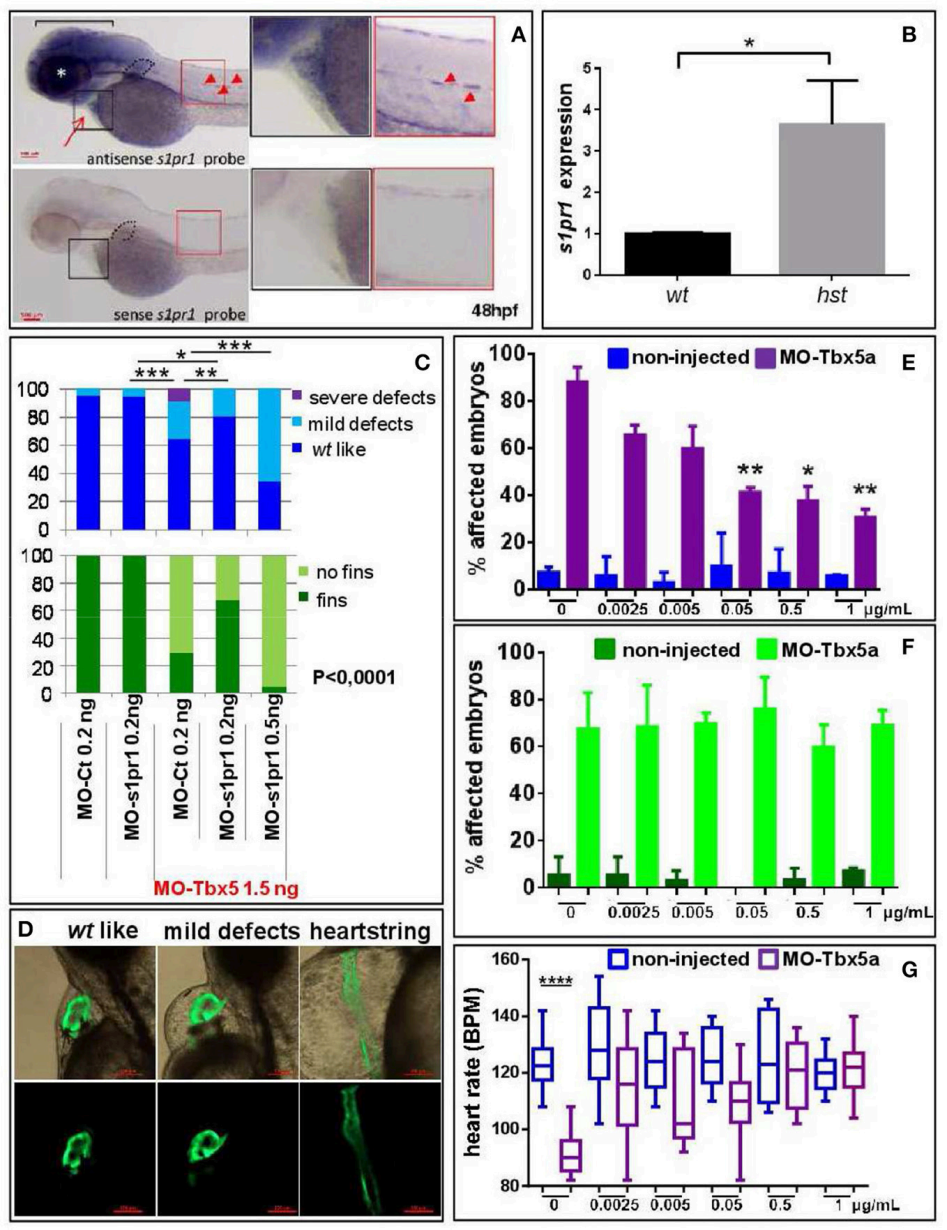
*s1pr1* is overexpressed in zebrafish HOS hearts and its downregulation is able to partially rescue heart/fin defects induced by *Tbx5* depletion. **(A)** Examples of *s1pr1* ISH performed on 48 hpf embryos. Top left, arrow and arrowheads indicate respectively the hybridization signals at the heart and vascular levels; dotted line encircles fin bud, bracket and asterisk mark respectively the hybridization signal on brain and eye areas Bottom left, control ISH performed with sense *s1pr1* probe. Right, magnifications of the cardiac and dorsal areas. Scale bar = 100 μm. **(B)** Quantification by ddPCR of *s1pr1* transcript in hearts dissected from 72 hpf normal and *tbx5a*^296^ mutant embryos. Data were normalized on ef1α as internal standard and are relative to *wt* sets as 1. Three different experiments starting from 3 different clutches were used for this analysis. **(C,D)** Rescue of Tbx5 morphants by *s1pr1* depletion. **(C)** Analysis of 72hpf Tbx5 morphants. MO-s1pr1 or MO-Ct at the reported doses, were co-injected with 1.5 ng of MO-Tbx5a in *Tg(Myl7:EGFP)*embryos. For comparison the phenotypes of embryos injected only with MO-s1pr1 or MO-Ct are presented. The percentage of embryos with the indicated heart (top) or pectoral fin (bottom) defects was averaged across multiple independent experiments carried out in double blind. All the comparisons indicated in the heart graph were highly significant in fin graphs (*P* < 0.0001). The total number of analyzed Tbx5 morphant embryos were as follows: MO-Ct co-injected *n* = 136 (0.2 ng), *n* = 137 (0.5 ng); MO-s1pr1 co-injected *n* = 136 (0.2 ng), *n* = 203 (0.5 ng). One hundred and fifty embryos were injected only with MO-Ct and 50 only with MO-s1pr1. **(D)** Some examples of the different cardiac phenotypes obtained in the experiments presented in **(C)**. *p < 0.05, **P < 0.001, ***P < 0.0001. **(E–G)** Rescue of Tbx5 morphants by *s1pr1* chemical inhibition. 2.5 ng of MO-Tbx5a was injected into *Tg(Myl7:EGFP)*embryos. At 24 hpf, W146 was added at the indicated doses to E3 medium of morphants and non-injected embryos. **(E)** Cardiac morphology analysis of 72 hpf Tbx5a morphants and non-injected embryos. Affected embryos either had heartstrings morphology, pericardial edema, or looping/chamber defects. The total number of analyzed Tbx5 morphant embryos were as follows: 0 μg *n* = 51, 0.0025 μg *n* = 32, 0.005 μg *n* = 27, 0.05 μg *n* = 29, 0.5 μg *n* = 34, 1 μg *n* = 32. The total number of analyzed non-injected control embryos were as follows: 0 μg *n* = 28, 0.0025 μg *n* = 38, 0.005 μg *n* = 30, 0.05 μg *n* = 31, 0.5 μg *n* = 31, 1 μg *n* = 34. *P < 0.05, **P < 0.001 vs. morphants with 0 μg/mL W146. None the non-injected control embryos treated with W146 showed any significant differences compared to embryos receiving 0 μg/mL W146. **(F)** Fin analysis of 72 hpf Tbx5a morphants and non-injected embryos. Fins were scored for the presence of both pectoral fins. No statistical differences were observed among drug application groups for either non-injected and Tbx5 morphant embryos, compared to no drug controls. **(G)** Heart rate analysis of 72 hpf Tbx5a morphants and non-injected embryos. Ten embryos were scored for each group as described in section Materials and Methods.

We have recently shown that Tbx5 depleted zebrafish embryos show a decreased level of miR-19a, and we demonstrated that miR-19a replacement partially rescues fin and cardiac defects caused by Tbx5 depletion (Chiavacci et al., 2015). Therefore we hypothesized that the miR-19a downregulation might cause an increase of *s1pr1* in heartstring (*hst*) embryos and that this increase might contribute to the HOS zebrafish phenotype. This hypothesis is supported by the observation that the phenotype of S1up embryos, including the altered heart morphology and the defective pectoral fins, was similar to the zebrafish mutant heartstrings (*hst* mut) (Garrity et al., 2002). To validate this hypothesis we first analyzed the *s1pr1* expression by checking the recently generated list of genes differentially modulated in *wt*- and Tbx5-depleted zebrafish embryos at 24 and 48 hpf (Table S1, D'Aurizio et al., 2016). The microarray data do not indicate significant variations of *s1pr1* expression in Tbx5-depleted compared with *wt* embryos at both developmental stages. However, *in situ* hybridization analysis performed in 48hpf embryos (Figure 6A) besides confirming the presence of *s1pr1* in fin buds and heart, shows a dominant *s1pr1* expression in nervous tissues. This high nervous expression might prevent a reliable detection of relatively low cardiac modulations in a whole embryo analysis. Therefore we decided to quantify the *s1pr1* expression in isolated hearts. This approach was performed by exploiting the Tbx5a^s296^ mutant line which demonstrates a G to A transition at base pair 527. This change creates a nonsense mutation at the amino acid 120 within the T-box region and is predicted to encoded a truncated protein, termed Tbx5a^s296^ (I. Scott and L. Parrie, unpublished data). Embryos homozygous for Tbx5a^s296^ develop the *hst* phenotype and lack any evidence of pectoral fins. Hearts were dissected from 72 hpf embryos obtained from crossing Tbx5a ^s296/+^parent line. We dissected hearts with the *hst* phenotype (from Tbx5a ^s296/s296^ embryos which lack pectoral fins) or with *wt* phenotype (from Tbx5a ^s296/+^ or Tbx5a^+/+^ siblings showing normal fins) as controls. Total RNA was extracted from *hst* and normal sibling hearts and the level of *s1pr1* transcript was analyzed by digital droplet PCR (ddPCR; Data Sheet 2). This analysis, which allowed us to accurately quantify a specific transcript, shows a 3-fold increase of *s1pr1* cardiac expression in *hst* compared to normal hearts (Figure 6B). Next, to verify if this increase could contribute to the HOS phenotype we performed rescue experiments. Figures 6C,D shows that co-injection of 1.5 ng of MO-Tbx5a with 0.2 ng of the MO-s1pr1 but not with the same quantity of the control morpholino, resulted in significantly fewer cardiac and fin defects. Injection of 0.2 ng of MO-s1pr1 alone does not affect embryo morphology to the degree that the 0.5 ng dose does (not shown); however higher dosage of MO-s1pr1 worsens the morphological alterations of HOS embryos (Figure 6C) suggesting that defined dosage of *s1pr1* is essential for the correct development of the heart.

To further support a role of *s1pr1* in Tbx5 regulatory circuit we treated Tbx5a morphants or non-injected embryos with the specific *s1pr1* inhibitor (R)-3-amino-4-(3-hexylphenylamino)-4-oxobutylphosphonic acid trifluoroacetate (W146) (Tarrason et al., 2011; Kunkel et al., 2013) or only with its vehicle. We used a large range of doses for this approach in light of the fact that there are no data concerning the use of W146 in zebrafish. However a progressive and dose dependent increase of vascular defects which we observed in zebrafish embryos was in accord with the *s1pr1* antagonist role played by this drug (not shown). Injection of 2.5 ng/ml of MO-Tbx5 generated heart defects in ~90% of embryos. Morphant embryos exposed to 0.05 to 1 ug/ml of W146 demonstrated a statistically lower incidence of heart defects (Figure 6E). In contrast, the incidence of heart defects in non-injected control embryos exposed to W146 was low and did not differ from the no-drug controls. On the contrary none of the tested doses was able to cause fin rescue (Figure 6F). To further confirm the positive effect of W146 in the cardiac context we analyzed the heart rate. The Tbx5 morphants present a heart rate significantly lower compared to control embryos, which is consistent with Tbx5 mutants (Garrity et al., 2002). While the lowest doses of W146 (0.0025–0.05 ug/ml) produced a variable increase in heart rate that did not reach statistical significance, the two higher doses of 0.5 or 1 ug/ml consistently restored the heart rate of Tbx5 morphants to statistically normal levels (Figure 6G). As expected, in the non-injected controls no heart rate differences were observed for any dose of W146.

Overall these data support the hypothesis that one of the negative consequences of Tbx5a depletion in zebrafish is an increase of *s1pr1* expression as consequence of miR-19a downregulation.

## Discussion

In this study we have uncovered a role of *s1pr1* in zebrafish cardiac development. Data obtained from *s1pr1* loss-of-function studies in zebrafish are rather contradictory (Ben Shoham et al., 2012; Gaengel et al., 2012; Tobia et al., 2012; Mendelson et al., 2013; Hisano et al., 2015). To investigate the role of this receptor in zebrafish we performed gain of function experiments overexpressing *s1pr1* to overcome both the possible functional redundancy among the s1P receptor family members and the concern about the use of morpholinos in this context.

Overexpression of *s1pr1* causes defects in ISV morphology and CVP confirming the contribution of this receptor to vascular patterning, but also generates incorrect cardiac looping and alterations of cardiac chamber morphology demonstrating a previously unappreciated role of *s1pr1* in heart development. At the higher dosages of injected *s1pr1*, the presence of a few cases of *cardia bifida* suggest its possible involvement in cardiac precursor migration through mechanisms which would be interesting to investigate. We observed a slight but significant upregulation of *s1pr2* in hearts isolated from *s1pr1* overexpressing hearts compared to control hearts. Although *s1pr2* mutation/downregulation is known to prevents cardiac precursors migrating thus resulting in *cardia bifida* (Kupperman et al., 2000), there are no data about the consequences of *s1pr2* upregulation. Therefore at the moment we can only hypothesize that *s1pr2* upregulation might contribute to the cardiac defects observed in *s1pr1* overexpressing embryos.

Disruption of the vascular network frequently causes circulatory defects and defective circulation can be cause of pericardiac edema and abnormal heart looping. Although we cannot exclude that in *s1pr1* overexpressing embryos circulation defects could, at some level, influence cardiac morphology, some considerations support a direct role of *s1pr1* in the cardiac context. First, we showed that overexpression of *s1pr1* influences the myocardial precursor pool before heart tube formation (Figures 3B,C). Moreover, low *s1pr1* dosages which do not affect vascular development and do not alter circulation, are nevertheless able to affect cardiac morphology (Figures 1, 2). Lastly, injection of the morpholino against *s1pr1* (MO-s1pr1) which strongly affects blood flow (as also reported in several papers), has minimal effects on heart development.

Interestingly, our analysis also highlights that *s1pr1* is subject to a negative post-transcriptional control by miR-19a. This effect has some important implications. The first one is that the activity of this receptor is hampered in the cardiac and vascular contexts where miR-19a is expressed, at least during the first stages of zebrafish development. In line with this observation a further decrease of *s1pr1* levels by MO-s1pr1 injection has a very limited impact on cardiac development (Figure S1) although it is known to strongly alter vascular morphology (Ben Shoham et al., 2012; Gaengel et al., 2012; Tobia et al., 2012; Mendelson et al., 2013). The role of miR-19a in *s1pr1* regulation has been functionally demonstrated: (1) by the observation that microinjections of the same quantities of *s1pr1* mRNAs have significantly different impacts on cardiovascular development depending on whether the mRNA includes its 3′UTR or not (Figure 2); (2) by showing that miR-19a gain or loss of function is able to respectively decrease or increase cardiovascular defects generated by *s1pr1* over-expression, only when its full length mRNA sequence has been injected. These findings corroborate the importance and the pervasiveness of microRNA-mediated regulatory control in the cardiovascular context. They also highlight the importance of transfecting the full length mRNA sequence of a gene under study to obtain a faithful picture of its biological impact, rather than injecting only the CDS sequence as was described for prior gain of function experiments performed with *s1pr1* (Mendelson et al., 2013).

The second important consequence of the post-transcriptional control of *s1pr1* by miR-19a is that since this microRNA is regulated by Tbx5 in zebrafish embryos (Chiavacci et al., 2015) then *s1pr1* is indirectly under the Tbx5 control and may contribute to the zebrafish HOS phenotype caused by Tbx5 depletion. The engagement of *s1pr1* in the Tbx5 regulatory circuit is supported by data showing an increase of this receptor in zebrafish Tbx5a mutant embryos and by rescue experiments. Both *s1pr1* depletion or inhibition by respectively morpholino or antagonist administration are able to decrease the level of cardiac defects. However while MO-s1pr1 injection positively impacts also fin morphology, this effect was not observed as a consequence of antagonist exposure. At the moment we have no explanation for this difference and we can only hypothesize that different tissue context might account for differences in drug susceptibility and/or toxicity.

Very recently, two new frame shift Tbx5 alleles have been generated using CRISPR-Cas9 mutagenesis. These mutants show the heart and fin defects which characterize *hst* mutants and morpholino knockdown, but do not present the strong heartstrings phenotype (Chiavacci et al., 2017). In light of these data which underlines the importance of analyzing several individual alleles of a candidate gene to evaluate its functions, it will be interesting to evaluate the impact of miR-19a and *s1pr1* modulation in all these different classes of mutants.

Our results, such as many others concerning *s1pr1*, are in contrast with data showing the absence of a clear phenotype in zebrafish mutants for this receptor (Hisano et al., 2015). However differences between the phenotypes caused by genetic mutations and those caused by gene knockdowns have been reported mainly in zebrafish where the use of reverse genetics is dramatically increased (Kok et al., 2015), but also in other model systems. While at the beginning these differences were interpreted as off target consequences of morpholino activity, more recently several experimental data point to the activation of compensatory networks. These networks are able to counterbalance deleterious mutations but appear not to be induced in response to translational or transcriptional knockdown (Rossi et al., 2015). The molecular mechanisms responsible of the different responses of mutants vs. morphants is object of debate, although a nonsense-mediated mRNA decay (NMD) activated by sequence alteration is suggested as a possible mechanism of induction (Rossi et al., 2015). In the case of *s1pr1*, the existence of several s1pr members with partial functional redundancy might offer an easy context for molecular compensation.

## Ethics statement

The corresponding author declares that all experiments were performed in accordance with relevant guidelines and regulations. The zebrafish facility, where all the experiments have been performed, is part of the CENTRO di BIOMEDICINA SPERIMENTALE (CBS) of the Area della Ricerca del CNR, Via Moruzzi 1, 56124 Pisa. The zebrafish facility has been authorized by the Italian Ministry of Health with the authorization n°297/2012-A of the 12/21/2012. The corresponding author declares that all the methods were carried out in accordance with the approved guidelines and that all experimental protocols were approved by the Italian Ministry of Health. Moreover all the technicians and researcher who take care of the animals and perform the experiments are appropriately trained by attending specific courses.

## Author contributions

LP designed the experiments; EG performed the most part of the microinjection experiments in zebrafish and the ISH; EC performed a minor part of the microinjection experiments in zebrafish; NA performed heart dissectioning of *hst* mutants and the ddPCR analysis; CI performed confocal imaging; MR performed QRT analysis; ME and LM realized the experiments on cell culture; LP wrote the manuscript; DG and FC revised the manuscript. MR and FC financed the work. All authors read and approved the final version of the paper.

### Conflict of interest statement

The authors declare that the research was conducted in the absence of any commercial or financial relationships that could be construed as a potential conflict of interest.

## Supplementary material

Data Sheet 1 Original Q-RT-PCR data relative to the analysis presented in Figure 3A.

Data Sheet 2 Original ddPCR data relative to the analysis presented in Figure 6B.

## Abbreviations

tbx5: T-box transcription factor 5
*hst*: *hearstring*
HOS: Holt-Oram syndrome
S1P: shingosine-1-phosphate
*s1pr1*: sphingosine-1-phosphate receptor 1
GPCRs: G protein-coupled receptors
*wt*: wild type
CDS: coding sequence
UTR: untranslated region
Scr: scramble
MO: morfolino
ef1α: elongation factor 1α
nppa: natriuretic peptide A
hand2: heart and neural crest derivates expressed 2
mef2ca: myocyte enhancer factor 2ca
mef2aa: myocyte enhancer factor 2aa
cx43: connection 43
cyp26b1: cytochrome P450, family 26, subfamily b, 1
gata4: GATA binding protein 4
cmlc2: cardiac myosin light chain 2
vmhc: ventricular myosin heavy chain
ISV: intersegmental vessel
CVP: caudal vein plexus
hpf: hours post fertilization
RFP: red fluorescent protein
GFP: green fluorescent protein
ddPCR: digital droplet PCR
RT-PCR: Real time PCR.
