# Supplementary material for: Post-transcriptional Modulation of Sphingosine-1-Phosphate Receptor 1 by miR-19a Affects Cardiovascular Development in Zebrafish
Source: Front Cell Dev Biol. 2018 Jun 5;6:58. doi: 10.3389/fcell.2018.00058 (PMC5996577; doi:10.3389/fcell.2018.00058)
Supplement: Data Sheet 1 — Original Q-RT-PCR data relative to the analysis presented in Figure 3A. [file Data_Sheet_1.zip › Caption data sheet 1.docx]

Original Q-RT-PCR data relative to the analysis presented in figure 3A.
