# Supplementary material for: Post-transcriptional Modulation of Sphingosine-1-Phosphate Receptor 1 by miR-19a Affects Cardiovascular Development in Zebrafish
Source: Front Cell Dev Biol. 2018 Jun 5;6:58. doi: 10.3389/fcell.2018.00058 (PMC5996577; doi:10.3389/fcell.2018.00058)
Supplement: Supplementary file 3 [file Image_1.PDF]

Supplementary Material
Post-Transcriptional Modulation of Sphingosine-1-Phosphate Receptor 1 by miR-19a Affects Cardiovascular Development in Zebrafish

Elena Guzzolino, Elena Chiavacci, NehaAhuja, Monica Evangelista, Chiara Ippolito, Deborah Garrity, Federico Cremisi and Letizia Pitto,*
* **Correspondence:** Corresponding Author*: l.pitto@ifc.cnr.it*

1.1 Supplementary Figures

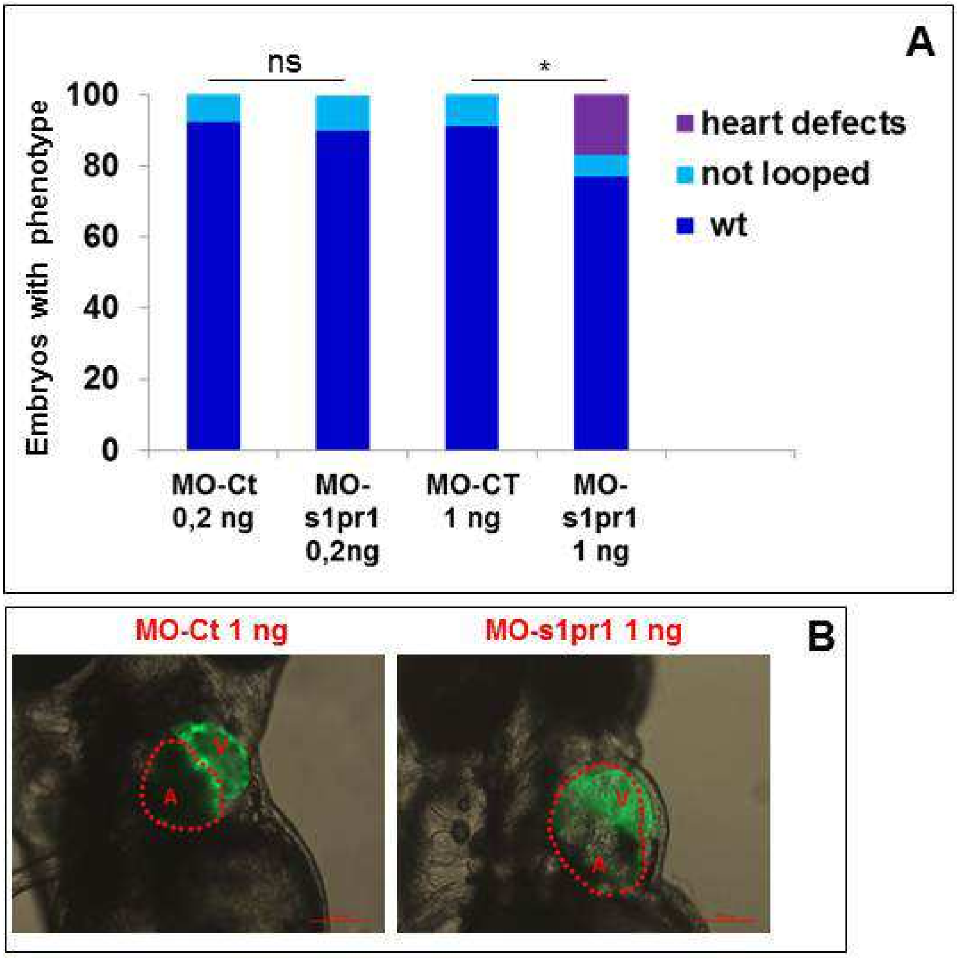

Figure S1. *s1pr1* downregulation has a small impact on heart development. (A) Analysis of 72hpf *Tg(Myl7:EGFP)* embryos injected with MO-s1pr1 or *MO*-Ct at the reported doses (ng). The percentage of embryos with the indicated heart defects was averaged across multiple independent experiments carried out in double blind. The total number of analyzed embryos were as follows: MO-Ct (0,2ng) n=152, MO-s1pr1 (0.2ng) n=146, MO-Ct (1ng) n=101, MO-s1pr1 (1 ng) n=117. T-test p= 0,014. (B) images are representative of hearts showing an enlarged atrium as a consequence of 1 ng MO-s1pr1 injection. Spotted lines highlight the atrium shape. V= ventricle A= atrium .Red scale bar = 100µm.
