# Supplementary material for: Post-transcriptional Modulation of Sphingosine-1-Phosphate Receptor 1 by miR-19a Affects Cardiovascular Development in Zebrafish
Source: Front Cell Dev Biol. 2018 Jun 5;6:58. doi: 10.3389/fcell.2018.00058 (PMC5996577; doi:10.3389/fcell.2018.00058)
Supplement: Supplementary file 4 [file Image_2.PDF]

Supplementary Material

Post-Transcriptional Modulation of Sphingosine-1-Phosphate Receptor 1 by miR-19a Affects Cardiovascular Development in Zebrafish

Elena Guzzolino, Elena Chiavacci, NehaAhuja, Monica Evangelista, Chiara Ippolito, Deborah Garrity, Federico Cremisi and Letizia Pitto,*
*** Correspondence:** Corresponding Author*: l.pitto@ifc.cnr.it*

1.2 Supplementary Figures

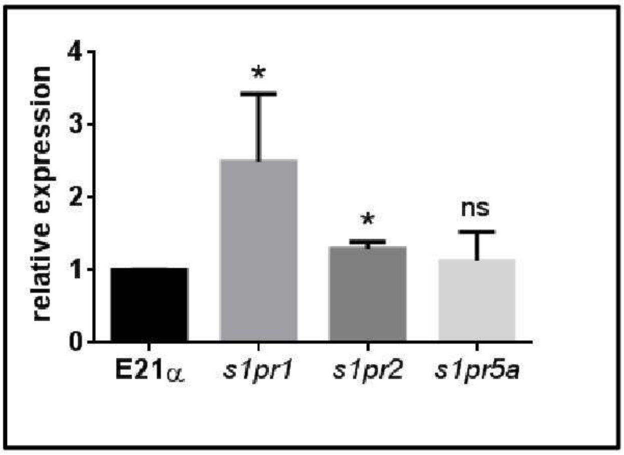

Figure S2. *s1pr1* overexpression slightly affects the expression of other cardiac members of *s1pr* family in zebrafish. One cell stage embryos were microinjected with 200 ng of the CDS mRNA of *s1pr1* or *RFP.* 48hrs after microinjection 20-30 hearts for each experimental group were dissected as described in Methods section. Total RNA was extracted, reverse-transcribed and Q-RT PCR performed. Data were normalized using *ef1α* as internal standard and are relative to values of hearts from RFP injected embryos. Three different experiments starting from 3 different clutches were used for this analysis.*P<0,05,
